# Abnormal hubs in global network as neuroimaging biomarker in right temporal lobe epilepsy at rest

**DOI:** 10.3389/fpsyt.2022.981728

**Published:** 2022-07-27

**Authors:** Ruimin Guo, Yunfei Zhao, Honghua Jin, Jihua Jian, Haibo Wang, Shengxi Jin, Hongwei Ren

**Affiliations:** ^1^Department of Medical Imaging, Tianyou Hospital of Wuhan University of Science and Technology, Wuhan, China; ^2^Key Laboratory of Occupational Hazards and Identification, Wuhan University of Science and Technology, Wuhan, China; ^3^Department of Neurosurgery, Tianyou Hospital of Wuhan University of Science and Technology, Wuhan, China; ^4^Department of Medical Imaging, Renmin Hospital of Wuhan University, Wuhan, China

**Keywords:** temporal lobe epilepsy, degree centrality, magnetic resonance imaging, receiver operating characteristic, caudate

## Abstract

While abnormal neuroimaging features have been reported in patients suffering from right temporal lobe epilepsy (rTLE), the value of altered degree centrality (DC) as a diagnostic biomarker for rTLE has yet to be established. As such, the present study was designed to examine DC abnormalities in rTLE patients in order to gauge the diagnostic utility of these neuroimaging features. In total, 68 patients with rTLE and 73 healthy controls (HCs) participated in this study. Imaging data were analyzed using DC and receiver operating characteristic (ROC) methods. Ultimately, rTLE patients were found to exhibit reduced right caudate DC and increased left middle temporal gyrus, superior parietal gyrus, superior frontal gyrus, right precuneus, frontal gyrus Inferior gyrus, middle-superior frontal gyrus, and inferior parietal gyrus DC relative to HC. ROC analyses indicated that DC values in the right caudate nucleus could be used to differentiate between rTLE patients and HCs with a high degree of sensitivity and specificity. Together, these results thus suggest that rTLE is associated with abnormal DC values in the right caudate nucleus, underscoring the relevance of further studies of the underlying pathophysiology of this debilitating condition.

## Introduction

Temporal lobe epilepsy (TLE) is a chronic neurological disease that can arise in response to a range of factors (1). Repeated abnormal epileptic discharges in affected patients can contribute to neuronal degeneration and necrotic death, in turn contributing to impaired cognition and altered brain tissue structural characteristics (2, 3). These structural changes in the brain of TLE patients can also disrupt normal memory and learning activity (4). TLE accounts for over 40% of all epilepsy cases and is the most common form thereof (5). However, at present, the diagnosis of epilepsy is largely dependent on a combination of medical history and EEG results. While EEG can accurately diagnose epilepsy in some cases, the successful recording of epileptic discharges generally only occurs in half of affected patients, and some healthy individuals may also exhibit false-positive EEG readings (6). As seizures occur without warning and are transient in nature, this can further complicate diagnostic efforts. Recent advances in neuroimaging technologies have led to a growing focus on the use of computer-aided imaging as a means of automating efforts to diagnose patients and localize lesions (7–9). Multimodal neuroimaging efforts can also clarify changes in the brains of TLE patients, providing new insight into the pathogenesis of this disease while aiding in efforts to predict and diagnose affected individuals (9).

Several different non-invasive, high-resolution neuroimaging modalities have been leveraged to aid in the diagnosis of specific neurological conditions. To date, several different functional magnetic resonance imaging (fMRI) studies have reported detecting a distinct blood oxygen level-dependent (BOLD) signal in brain regions where seizure foci are located (10, 11). These fMRI techniques enable the effective localization of epileptic lesions with a combination of high temporal and spatial resolution (12). Reduced regional signal in the right posterior cingulate cortex and precuneus (PCu) regions, for example, has been identified as a biomarker characteristic of patients with epilepsy (13, 14). Moreover, altered amplitude of low-frequency fluctuation (ALFF) values in particular regions of the brain can reliably differentiate between epilepsy patients and healthy controls (HCs) with high levels of sensitivity and specificity (15, 16). Structural MRI studies have revealed that several brain regions in patients with epilepsy also exhibit structural abnormalities detectable through neuroimaging (17). However, no simple and accurate neuroimaging biomarker capable of guiding the early detection of TLE has yet been established.

Degree centrality (DC) is a whole-brain connectivity index that describes the global features of a given node through the use of graph theory models to assess the functional connectivity between that node and nodes throughout the brain (18). DC-based analytical approaches have recently been used to successfully evaluate patients diagnosed with schizophrenia, depression, and mild cognitive impairment (19–21). Moreover, the combination of DC and machine learning algorithms can predict responses to treatment in children with epilepsy undergoing antiepileptic medication treatment (22). Increases in voxel-wise DC values in specific brain regions correspond to an increased degree of global connectivity, whereas reductions in voxel-wise DC values are indicative of a reduction in the degree of global connectivity. Studies of voxel-based DC have been used to analyze altered brain functionality in patients with epilepsy at the whole-brain level, with one recent report having revealed the existence of abnormal DC findings in the medial superior frontal gyrus (MSFG), left dorsolateral superior frontal gyrus, right inferior parietal lobule, and the left postcentral region of adults with epilepsy (9). Therefore, it is necessary to combine DC and operating characteristic (ROC) methods when exploring imaging biomarkers of right temporal lobe epilepsy (rTLE).

The present study was developed with the goal of assessing whether rTLE patients exhibit abnormal DC activity, with the hypothesis that these abnormal DC values could be utilized as neuroimaging biomarkers to reliably distinguish between rTLE patients and HC individuals.

## Materials and methods

### Participants

In total, this study enrolled 68 patients with rTLE diagnosed as per the criteria established by the International Anti-Epilepsy League that were recruited from Tianyou Hospital Affiliated to Wuhan University of Science and Technology. In addition, 73 age- and sex-matched HC individuals were recruited from among patients at Tianyou hospital undergoing routine physical examinations. To be eligible for study inclusion, rTLE patients had to meet the following criteria: (1) patients exhibited typical TLE symptoms suggestive of the presence of epileptic foci in the right temporal lobe; (2) patients exhibited interictal EEG results consistent with the potential presence of right temporal lobe lesions; and (3) patients exhibited MRI results indicative of right hippocampal atrophy or sclerosis. Patients were excluded if they were left-handed or had any history of traumatic brain injury, psychiatric disease, or other neurological diseases. All participants provided written informed consent. The medical ethics committee of Tianyou Hospital Affiliated to Wuhan University of Science and Technology approved this study, which was consistent with the Declaration of Helsinki.

### Magnetic resonance imaging acquisition and processing

A 3.0T Philips MRI instrument was used to collect all resting-state fMRI data at Tianyou Hospital. Participants were directed to remain awake with their eyes closed during imaging. All echoplanar imaging sequences were acquired with the following settings: repetition time/echo time (TR/TE) 2000/30 ms, 31 slices, 220 × 220 matrix, 90° flip angle, 24 cm field of view, 5 mm thick layers without gaps. The processing of all fMRI data was performed using the Data Processing Assistant for rs-fMRI, which functions with SPM12 implemented in MATLAB. The first 10 images for each participant were excluded from the analysis to allow for patient adaptation and to mitigate the potential effects of initial MRI signal instability. Participants were excluded if they exhibited maximum displacement >2 mm in the *x*-, *y*-, or *z*-axis directions or >2° of maximum angular rotation after correction for head movement and slice timing. The corrected imaging data were spatially normalized to the MNI space and resampled at 3 mm × 3 mm × 3 mm, after which they were subjected to temporal bandpass filtering (0.01–0.08 Hz) and linear detrending. Certain spurious covariates were removed from these images, including signals from ventricular seed-related regions and white matter central regions, as well as 24 head motion parameters derived from rigid body correction. Global signals were preserved when processing resting-state functional connectivity data, as in prior reports (23).

### Degree centrality calculation

Degree centrality measurements were made using the REST-DC toolkit from the REST package^1^. Pearson correlation coefficients were used to compute DC values, with Pearson correlation coefficients corresponding to the relationship between all voxel pairs in a time series being used to establish a graph for each study participant. In this graph, individual brain voxels are regarded as nodes, while significant correlations between nodes are regarded as edges. The n*n Pearson correlation coefficient matrix between each pair of voxels was then utilized to establish subjective whole-brain functional connectivity. To enhance the normalization of these data, they were transformed into a *Z*-score matrix by applying Fisher’s *R*-to-*Z* transformation, with the sum of *Z* values between selected voxels and all other voxels being used to generate weighted voxel DC values. To eliminate any potential false connectivity, a Pearson correlation coefficient threshold of *r* > 0.25 was established by thresholding each correlation at *P* < 0.01.

### Statistical analysis

Data were analyzed using SPSS 22.0 (SPSS Inc., IL, United States). Demographic differences were compared between groups using two-sample *t*-tests and chi-square tests.

### Correlation analysis

Degree centrality values were extracted from brain regions that exhibited abnormalities between these two groups, after which Pearson correlation coefficients were used to detect the relationships between these abnormal DC values and clinical variables of interest.

## Results

### Demographics and clinical features

Participant demographic and clinical characteristics are shown in Table 1. There were no significant differences in age, sex, or education level between rTLE patients and HCs (*P* > 0.05).

**TABLE 1:** Demographic information.

Characteristics	Patients (*n* = 68)	Controls (*n* = 73)	*x*^2^ or *T*	*P* value
Gender (male/female)	68 (35/33)	73 (37/36)	0.67	0.06^a^
Age (years)	28.59 ± 6.09	28.30 ± 4.24	0.34	0.73^b^
Education (years)	12.12 ± 2.43	12.01 ± 2.70	1.77	0.19^b^
Illness duration (months)	8.35 ± 2.93			

^a^The *p* value for gender distribution was obtained by chi-square test.

^b^The *p* value were obtained by two sample *t*-tests.

### Degree centrality differences

**FIGURE 1:**
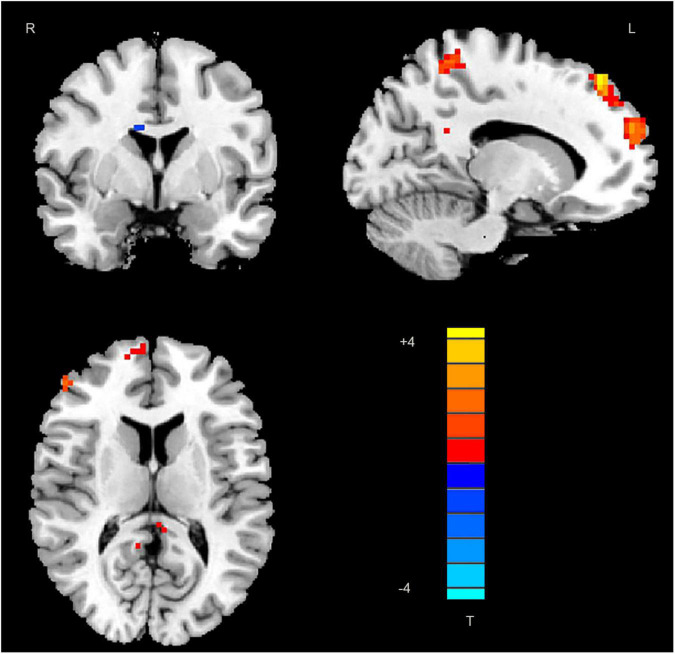
Decreased DC values in the right caudate and increased DC values in the left middle temporal gyrus, superior parietal gyrus, superior frontal gyrus, right precuneus, frontal gyrus Inferior gyrus, middle-superior frontal gyrus, and inferior parietal gyrus in patients. DC, degree centrality; MTG, middle temporal gyrus; SPG, superior parietal gyrus; SFG, superior frontal gyrus; PCu, precuneus; IFG, frontal gyrus Inferior gyrus; MSFG, middle-superior frontal gyrus; and IPG, inferior parietal gyrus.

Significant differences in DC values in different regions of the brain were next identified by comparing data from rTLE patients and HCs. Relative to HC individuals, rTLE patients exhibited significantly reduced DC values in the right caudate gyrus, as well as significantly increased DC values in the left middle temporal gyrus (MTG), superior parietal gyrus (SPG), superior frontal gyrus (SFG) and right PCu, inferior frontal gyrus (IFG), MSFG, and inferior parietal gyrus (IPG; Figure 1 and Table 2). There were no significant correlations between any of the identified DC values and rTLE patient disease duration.

**TABLE 2:** Alterations of DC between patients and controls.

Cluster location	Peak (MNI)			Number of voxels	*T*-value	*P*
	*X*	*Y*	*Z*			
Left MTG	−48	−42	−9	40	3.48	<0.01
Right PCu	6	−48	18	111	3.69	<0.01
Right IFG	51	42	6	30	3.49	<0.01
Right MSFG	9	63	24	67	3.94	<0.01
Right IPG	39	−45	45	30	3.33	<0.01
Right caudate	12	−3	27	47	−4.02	<0.01
Left SFG	−12	60	21	66	3.55	<0.01
Left SPG	−18	−42	63	70	3.57	<0.01

DC, degree centrality; MTG, middle temporal gyrus; PCu, precuneus; IFG, inferior frontal gyrus; MSFG, medial superior frontal gyrus; IPG, inferior parietal gyrus; and SFG, superior parietal gyrus.

### Receiver operating characteristic results

**FIGURE 2:**
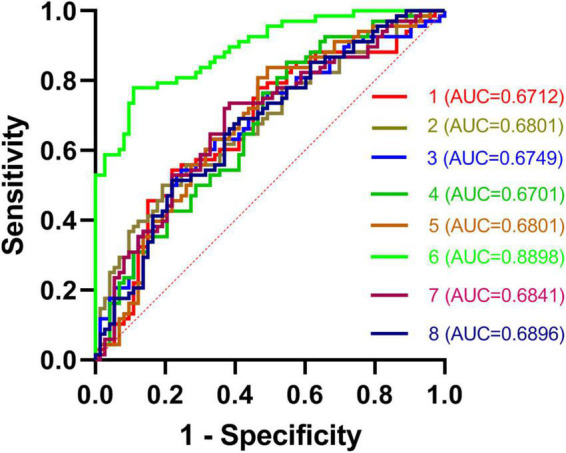
ROC curves for the differentiation between TLE patients and HCs based on DC values in abnormal brain regions. TLE, temporal lobe epilepsy; ROC, receiver operating characteristic; HCs, healthy controls; and DC, degree centrality.

In total, rTLE patients exhibited abnormal DC values in 8 regions of the brain (right caudate, left MTG, SFG, SPG, right PCu, IFG, MSFG, and IPG). Subsequent ROC analyses indicated that abnormal DC values in the right caudate nucleus exhibited the highest AUC value (0.8898; Figure 2).

## Discussion

Here, DC values were used to differentiate between rTLE patients and HCs. Overall, patients diagnosed with rTLE were found to exhibit reduced DC values in the right caudate with corresponding increases in DC values in the left MTG, SPG, SFG, and right PCu, IFG, MSFG, and IPG relative to HCs. The abnormal DC values in the right caudate nucleus were able to effectively discriminate between these two participant groups with an AUC value of 0.8898.

Receiver operating characteristic analyses are commonly used for the diagnosis of neuropsychiatric disorders such as schizophrenia or major depression (24–26). To be reliable, a diagnostic indicator must exhibit an ROC value of 0.6 or higher (27). The AUC value derived from the ROC analysis of the right caudate nucleus was higher than that for any other tested regions exhibiting DC abnormalities between rTLE patients and controls. The caudate nucleus is an important mediator of cognition, and certain neurological disorders have been shown to differentially impact the dorsal and ventral caudate nuclei (28). Both functional activity and connectivity can be used to subdivide the caudate into these ventral and dorsal regions, with the dorsal caudate nucleus being closely associated with the dorsolateral prefrontal cortex and shaping executive function and working memory (29, 30). In contrast, the ventral caudate nucleus is more closely linked to the limbic system and to pain processing and other emotional functions. Many studies have evaluated the caudate nucleus in patients (31, 32). Here, right caudate nucleus DC values were significantly reduced in rTLE patients relative to HCs, with the connection strength between this region and other regions of the brain thus being reduced, potentially explaining the observed declines in learning, attention, and memory that these patients experience. However, these reduced DC values were not correlated with patient symptom severity or disease duration, suggesting that they are independent of disease severity in rTLE patients. Notably, this DC reduction in the right caudate nucleus was able to readily differentiate between rTLE patients and HCs, suggesting that this change is characteristic of this form of epilepsy.

The frontal lobe is a key mediator of cognitive control, with abnormalities in the structure and function of the frontal lobe being reported in prior studies of patients with TLE (33, 34). Patients exhibiting cognitive impairments including decreased language fluency and processing speed have been reported relative to HCs, as has a case of patients with notably poor cognitive function. Here, abnormal DC values in the MFG were observed in rTLE patients, potentially partially accounting for the cognitive deficits that TLE patients experience.

Many structural and functional studies of TLE patients focus on the temporal lobe, including the amygdala, HIC, parahippocampal gyrus, and entorhinal cortex (1). The MTG is an important component of semantic memory and language processing-related activity (35, 36). Recently, one study found that TLE was associated with alterations in the intrinsic connectivity of both temporal regions (37). The temporal lobe is also closely related to social cognition and emotional processing (4). Moreover, the MTG is a key node in a broad network of frontal, parietal, occipital, and subcortical structures, with abnormal MTG activation thus potentially being an important pathophysiological component of rTLE-related brain damage (38). Abnormal MTG activity may also impact temporal lobe function given its role as a regulator of language processing and semantic memory. Observed increases in DC in the MTG in rTLE patients may thus be linked to cognitive deficits, although memory testing was not employed in the present study.

The parietal lobe is closely related to key cognitive and sensory functions including memory, attention, motor learning, spatial perception, and visuomotor integration (39, 40). Anatomical and functional characteristics are used to subdivide the parietal lobe into five regions, two of which are related to motor processes and visual orientation, whereas the remaining three are associated with reasoning, working memory, visual perception, attention, and spatial cognition (41). The SPL region has been specifically linked to interaction and locomotion in macaques. Adult TLE patients exhibt increases in SPL density detected through structural MRI and fMRI studies (42). The SPL and the associated intraparietal groove are important in the context of multi-object tracking as analyzed based on changes in BOLD signal, with both of these regions being related to sustained attention and object indexing (43). The SPL additionally interacts with the prefrontal cortex to coordinate stimulus-oriented processes and executive function, and correlates with inhibition in the left inferior parietal cortex (44). Neuroimaging analyses have demonstrated a relationship between decreased visual acuity and the bilateral SPL (43). The contralateral posterior parietal cortex, ipsilateral inferior frontal gyrus, and ipsilateral frontal areas also exhibit more robust anatomical connections to the right posterior SPL rather than the left SPL. The results of this study suggest that increases in DC values in the SPL may be linked to the pathogenesis of rTLE.

The PCu is the master node of the default mode network, and is involved in the processing of visuospatial information, metaphors, mental imagery, and episodic memory (45). Structural MRI studies have revealed that significantly altered spontaneous brain activity in the bilateral PCu is linked to the pathophysiology of epilepsy and depression (46). Functional MRI analyses have further highlighted decreased activation clusters in the precuneus and supramarginal gyrus of TLE patients as compared to HCs, with positive correlations between conceptual creativity and the gray matter volume of the PCu (47). Enhanced PCu cortical surface area is also linked to morphological variations in the adult midsagittal brain. Recent work suggests that the PCu may also coordinate correlations between vivid memories and egocentric perspectives, with an increase in PCu volume being related to an increased tendency to recall egocentric episodic autobiographical memories (48). Reduced right PCu ReHo has been reported to be related to higher levels of verbal innovation capacity linked to improved flexibility, ingenuity, and fluency (49). These characteristics are common among TLE patients. One meta-analysis found PCu to be activated in response to familiarity (50). The PCu can also promote consciousness networks that exhibit selective hypometabolism in epilepsy patients (51). Here, enhanced PCu DC values in rTLE patients may thus represent a compensatory mechanism associated with the pathogenesis of this disease.

There are multiple limitations to this study. For one, patients were treated with antiepileptic drugs, which may have influenced the brain networks in these patients. However, it was not possible to exclude antiepileptic drug use from a patient care perspective. Second, this study had a limited sample size. Third, neuropsychological scale assessments for all patients were not available owing to patient reluctance to undergo these analyses.

In summary, these data suggest that rTLE patients exhibit abnormal DC values in several regions of the brain, with altered DC in the right caudate offering potential value as neuroimaging biomarker of rTLE.

## Data availability statement

The original contributions presented in the study are included in the article/supplementary material, further inquiries can be directed to the corresponding authors.

## Ethics statement

The studies involving human participants were reviewed and approved by the Medical Ethics Committee of the Tianyou Hospital Affiliated to Wuhan University of Science and Technology. The patients/participants provided their written informed consent to participate in this study.

## Author contributions

RG: writing the manuscript. YZ, HJ, and JJ: study conceptualization, organization, and analysis outcome verification. JJ and HW: data collection and analysis. HW, HR, and SJ: manuscript preparation. RG, HR, and SJ: collect pictures and process them. All authors contributed to the article and approved the submitted version.

## Footnotes

1
http://www.restfmri.net/
